# RAGE and HMGB1 Expression in Orbital Tissue Microenvironment in Graves' Ophthalmopathy

**DOI:** 10.1155/2021/8891324

**Published:** 2021-03-11

**Authors:** Dominika Łacheta, Krzysztof B. Poślednik, Katarzyna Czerwaty, Nils Ludwig, Marta Molińska-Glura, Ireneusz Kantor, Anna Jabłońska-Pawlak, Piotr Miśkiewicz, Alicja Głuszko, Zygmunt Stopa, Jacek Brzost, Mirosław J. Szczepański

**Affiliations:** ^1^Department of Biochemistry, Medical University of Warsaw, 02-097 Warsaw, Poland; ^2^Department of Otolaryngology, Medical Centre for Postgraduate Education, 03-242 Warsaw, Poland; ^3^Department of Oral and Maxillofacial Surgery, University Hospital Regensburg, 93053 Regensburg, Germany; ^4^Department of Forest Technology of Poznan University of Life Sciences, 60-624 Poznan, Poland; ^5^Department of Ophthalmology, Medical University of Warsaw, 02-097 Warsaw, Poland; ^6^Department of Internal Medicine and Endocrinology, Medical University of Warsaw, 02-097 Warsaw, Poland; ^7^Department of Maxillofacial Surgery, Medical University of Warsaw, 02-097 Warsaw, Poland; ^8^Department of Otolaryngology, The Children's Memorial Health Institute, 04-730 Warsaw, Poland

## Abstract

Graves' ophthalmopathy (GO) is a chronic autoimmune inflammatory disorder involving orbital tissues. A receptor for advanced glycation end products (RAGE) and its ligand high mobility group box 1 (HMGB1) protein trigger inflammation and cell proliferation and are involved in the pathogenesis of various chronic inflammatory diseases. This study was aimed to evaluate RAGE and HMGB1 expression in GO to determine its potential clinical significance. To the best of our knowledge, this is the first study showing RAGE and HMGB1 expression in orbital tissue using immunohistochemistry. Sections of orbital adipose tissue obtained from patients diagnosed with GO (23 patients; 36 orbits) and normal controls (NC) (15 patients; 15 orbits) were analyzed by immunohistochemistry for RAGE and HMGB1 expression. Expression profiles were then correlated with clinical data of the study group. RAGE and HMGB1 expression were elevated in GO patients in comparison with NC (*p* = 0.001 and *p* = 0.02, respectively). We observed a correlation between RAGE expression and occurrence of dysthyroid optic neuropathy (DON) (*p* = 0.05) and levels of TSH Receptor Antibodies (TRAb) (*p* = 0.01). Overexpression of RAGE and HMGB1 might be associated with GO pathogenesis. In addition, RAGE and HMGB1 proteins may be considered as promising therapeutic targets, but this requires further research.

## 1. Introduction

Graves' disease (GD) represents an autoimmune process in which circulating autoantibodies directed against thyrotropin receptor (TSHR)—TRAb (TSHR antibodies)—activate the thyroid gland, causing hyperthyroidism [1]. One of the extrathyroidal symptoms of GD is Graves' ophthalmopathy (GO), defined as a chronic autoimmune inflammatory disorder involving orbital tissues [2]. Although patients with GO are mostly hyperthyroid, they can also be euthyroid or hypothyroid. Moreover, GO may be reported in Hashimoto's thyroiditis [3]. Cytokine production, inflammatory infiltration, and orbital fibroblast activity result in expansion and remodeling of extraocular tissues—mainly orbital adipose tissue and fibrous tissue of extraocular muscles. Edematous-infiltrative changes involving orbital soft tissues are observed in 25–50% of patients with GD [4]. Clinical manifestations of GO include lid retraction, double vision, soft tissue swelling, and erythema of the conjunctival and periorbital tissues. Increased intraocular pressure within the inflexible bony orbital walls can contribute to the development of proptosis and optic nerve compression, including dysthyroid optic neuropathy (DON). According to the European Group on Graves' Orbitopathy (EUGOGO), severity of GO is rated as mild, moderate-to-severe, and sight threatening (including DON and/or severe keratitis) [5].

Multiligand receptor for advanced glycation end products (RAGE) is suggested to initiate and amplify immune and inflammatory responses [6]. Increased levels of RAGE ligands in chronic disorders indicate that RAGE is involved in the pathogenesis of various inflammatory diseases [7]. Cellular stress causes the generation of RAGE ligands such as high mobility group box 1 (HMGB1) protein, S100 proteins, and nucleic acids, while prolonged hyperglycemia and inflammation induce the release of the ligands AGE and amyloid [8]. HMGB1 is one of the most significant members of the DAMP (damage-associated molecular patterns) family. DAMPs involve molecules released by dying or necrotic cells and can induce inflammation, cell proliferation, and migration [9]. HMGB1-RAGE interaction affects inflammation via the activation of proinflammatory transcription factor NF-*κ*B (nuclear factor kappa B) which furthermore regulates RAGE [10].

In this study, we test the hypothesis that RAGE and HMGB1 are overexpressed in orbit tissue in GO and that expression patterns correlate with disease severity. To the best of our knowledge, this is the first study showing RAGE and HMGB1 expression in orbital tissue by immunohistochemistry.

## 2. Materials and Methods

### 2.1. Patients and Tissue Collection

Archival tissue paraffin blocks were used for the studies. All orbital adipose tissue samples used in the study were collected during surgery for routine histopathological examination between 2016 and 2020. Tissues were obtained from 23 patients (18 females, 5 males, 36 orbits) diagnosed with GO who underwent transnasal endoscopic orbital decompression surgery in the Otolaryngology Department, Centre of Postgraduate Medical Education in Warsaw, Poland, or were derived from a tissue bank at the Department of Internal Medicine and Endocrinology, Medical University of Warsaw, Poland, from previous studies. Inclusion criteria for the study were in accordance with the EUGOGO guidelines: (1) moderate-to-severe GO or (2) sight-threatening GO. Patients with any of the following conditions were excluded from the study: (1) age under 18 years, (2) a history of eye surgery, (3) a previous history of metabolic diseases or other diseases that may affect orbital connective tissues, except for thyroid disease, and (4) pregnancy or lactation. As controls, orbital adipose tissue samples were obtained from 15 patients (15 orbits) operated on the orbit due to trauma at the Department of Maxillofacial Surgery, Medical University of Warsaw, Poland. The study was approved by Local Ethics Committees at the Medical University of Warsaw in Poland (#AKBE/86/2018 to P.M.; KB/126/2016 to M.J.S) and at the Centre of Postgraduate Medical Education (#16/PB/2018 to I.K.). Tissues were fixed in 10% formaldehyde and embedded in paraffin. Sections were stained with hematoxylin-eosin (H+E) and evaluated by light microscopy before performing immunohistochemistry.

### 2.2. Clinical Characteristics of Graves' Ophthalmopathy

Ocular involvement, including GO severity and activity, was defined as absent, mild, moderate-to-severe, and sight-threatening—active or inactive, according to criteria reported in EUGOGO recommendations [5, 11]. Patients were classified as “active” or “inactive” based on a Clinical Activity Score (CAS). Dysthyroid optic neuropathy (DON) was defined by visual dysfunction secondary to GO when other causes for visual impairment had been excluded. The diagnosis of DON was based on at least two criteria from the following: (1) reduced visual acuity (<1.0), (2) relative afferent pupillary defect, (3) reduced color vision (more than two errors in Ishihara plates), (4) optic disc swelling in the affected eye, and (5) magnetic resonance imaging of orbit showing apical crowding or optic nerve stretching. Corneal involvement (keratitis) was defined as absent or punctuate keratopathy/ulcer. Best-corrected visual acuity (BCVA) was examined using Snellen charts and expressed as a decimal fraction. Exophthalmos was measured by a Hertel exophthalmometer. The intraocular pressure was measured in the primary position using an applanation tonometer. All the ophthalmology examinations for all patients were carried out by the same ophthalmologist. Clinicopathological characteristics of Graves' ophthalmopathy (GO) patients are presented in Table 1.

### 2.3. Immunohistochemistry

Paraffin sections of GO patients and NC patients were immunostained using NovoLink Polymer Detection Systems (Novocastra Laboratories, Newcastle, UK) and the following primary antibodies diluted 1 : 100 in Antibody Diluent (Dako): rabbit polyclonal anti-human HMGB1 (LS-C2691, LifeSpan BioSciences, Inc., Seattle, WA, USA) and mouse monoclonal anti-human RAGE (LS-B6042, LifeSpan BioSciences, Inc., Seattle, WA, USA). Deparaffinated and rehydrated sections were stained according to the manufacturer's instructions, as previously described [12]. The activity of endogenous peroxidase was blocked by Peroxidase Block (NovoLink Polymer Detection System; Novocastra Laboratories). Sections were incubated with primary antibodies overnight and were subsequently incubated with Post Primary (rabbit anti-mouse IgG; NovoLink Polymer Detection System; Novocastra Laboratories) which detected mouse antibodies and with Polymer (NovoLink Polymer Detection System; Novocastra Laboratories) to recognize rabbit antibodies and detect Post Primary and finally with 3,3′-diaminobenzidine chromogen (NovoLink Polymer Detection System; Novocastra Laboratories). Nonspecific binding of the primary antibodies and the polymer was eliminated by application of Protein Block (NovoLink Polymer Detection System; Novocastra Laboratories) before adding the primary antibodies. Sections were counterstained with hematoxylin (Dako), dehydrated, coverslipped, and evaluated by light microscopy ZEISS Observer Z1 (Axiovision 4.8 software; illumination system LUMEN 200; PRIOR) in high power field (magnification ×400). Results were scored by two independent investigators (M.J.S. and D.Ł.) as positive (++), heterogeneous (+), or negative (-), when the number of stained cells in each section was >75 cells, between 25 and 75 cells, and <25 cells, respectively, as previously described [13].

### 2.4. Statistical Analysis

Statistical analysis was performed by professional statistician using the Statistica 13 software package. The Fisher exact test was used for the binary variable. When comparing continuous variable, without normal distribution (checked with a Kolmogorov-Smirnov test), differences between groups were measured using Mann–Whitney's *U* test. The significance level was established at *p* ≤ 0.05.

## 3. Results

### 3.1. RAGE and HMGB1 Expression in Tissue Sections

**Figure 1:**
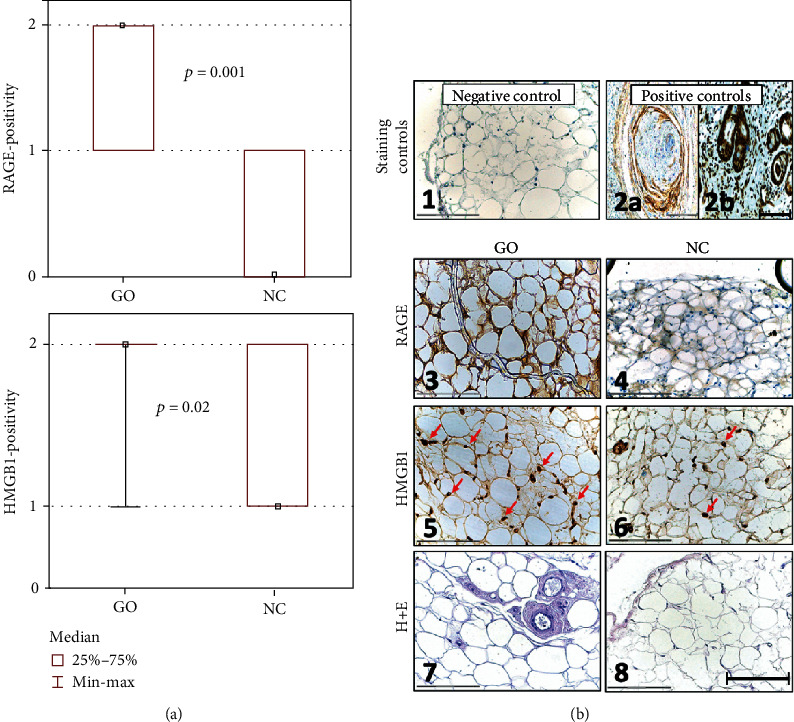
RAGE and HMGB1 expression in normal control (NC) and in Graves' ophthalmopathy (GO) tissues. **(**a) Statistical analysis of RAGE and HMGB1 expression in GO tissues compared to NC. The sections were scored as described in Material and Methods. (b) Representative images of immunohistochemistry staining. B1: isotype negative control of GO tissue. B2: positive control. (a) RAGE expression on head and neck squamous cell carcinoma tissue. (b) HMGB1 expression on nasal cavity mucosa. B3: RAGE expression in GO tissue. B4: RAGE expression in NC tissue. B5: HMGB1 expression in GO tissue (arrows). B6: HMGB1 expression in NC tissue (arrows). B7: hematoxylin and eosin (H+E) staining of GO tissue. B8: H+E staining of NC tissue. *p* value ≤ 0.05 was considered to be significant. Bar = 150 *μ*m.

RAGE expression levels were elevated in GO tissues compared to those from NC (*p* = 0.001; Figure 1(a)). In GO tissues, RAGE was detected in the cytoplasm, and the expression was positive or heterogenous with staining intensities ranging from weak to strong. In contrast, RAGE expression was observed in only 25% of NC tissues and its staining intensity ranged from negative to weak (Figure 1(a)).

HMGB1 was detectable in the nuclei and cytoplasm in all tissues of GO patients and NC (Figure 1(b)). Intensity of HMGB1 staining was evaluated as moderate or strong (Figures 1(a) and 1(b)). Differences between GO and NC in terms of HMGB1 expression were statistically significant (*p* = 0.02; Figure 1(a)).

### 3.2. RAGE Expression Correlates with Disease Severity

**Figure 2:**
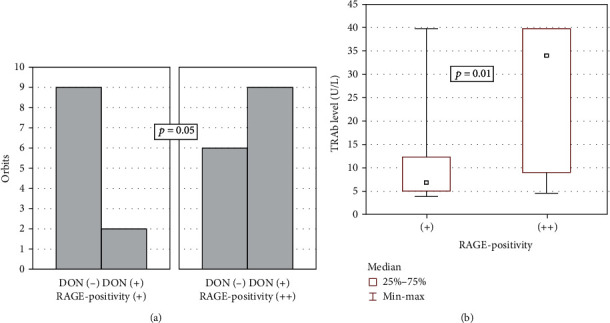
RAGE expression in correlation with clinical data. (a) RAGE positivity vs. occurrence of dysthyroid optic neuropathy (DON) (*p* = 0.05). (b) RAGE positivity vs. levels of TSHR antibodies (TRAb; *p* = 0.01). *p* value ≤ 0.05 was considered to be significant.

In the GO cohort, we observed differences in RAGE positivity depending on occurrence of DON and TRAb levels (Figure 2). GO patients with DON had stronger expression of RAGE (positivity “++”) than tissues from patients without DON (*p* = 0.05) (Figure 2(a)). Moreover, RAGE staining correlated with TRAb levels in GO patients. Positivity “++” was characteristic for high levels of TRAb (*p* = 0.02; Figure 2(b)).

The correlation of HMGB1 expression levels in the GO cohort with clinical data revealed no statistically significant differences (*p* > 0.05). However, we observed the tendency between increased levels of HMGB1 expression in patients with sight-threatening GO with DON (data not shown).

### 3.3. RAGE and HMGB1 Expression in Inflammatory Infiltrates

**Figure 3:**
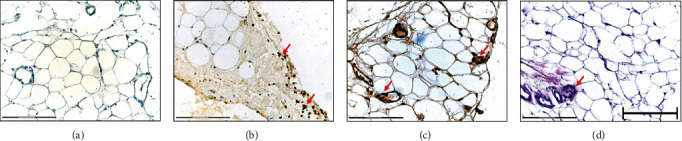
RAGE and HMGB1 expression on inflammatory infiltrates in GO tissues. (a) Isotype negative control staining of GO tissue. (b) RAGE expression on inflammatory infiltrates (arrows). (c) HMGB1 expression on inflammatory infiltrates (arrows). (d) H+E staining of GO tissue. Bar = 150 *μ*m.

In GO tissues, we observed elevated numbers of inflammatory cells that expressed RAGE and HMGB1 proteins. Those polymorphonuclear and mononuclear cells were mainly localized in close proximity to the vessels (Figure 3).

## 4. Discussion

In our study, we demonstrated significant differences between GO and NC tissues for RAGE and HMGB1 expression. Enhanced expression of RAGE occurs in conditions of inflammatory mediators and ligands for RAGE accumulation [8]. Literature data indicate that the inflammatory process comprising orbital tissues underlies the pathogenesis of GO [14, 15]. Immune cell infiltration as well as cytokine production activates orbital fibroblasts and GAG synthesis resulting in adipose tissue expansion. Yoon et al. have demonstrated that mRNA and protein levels of the proinflammatory cytokines TNF-*α* and IL-1*β* are increased in GO tissues compared to healthy controls [16]. Furthermore, our preliminary study has shown that a variety of proteins and cytokines involved in inflammation, autoimmunity, hypoxia, and fibrosis are expressed in GO orbital adipose tissue microenvironment, including TGF-*β*, TLR-4 (Toll-like receptor 4), HIF-1*α* (hypoxia-inducible factor-1*α*), NF-*κ*B (nuclear factor kappa B), and IL-17 [14].

Current evidences point out that HMGB1 protein is involved in numerous chronic inflammatory and autoimmune diseases including rheumatoid arthritis, atherosclerosis, and systemic lupus erythematosus (SLE) [17, 18]. Released HMGB1 interacts with its cell-surface receptor—RAGE, activating the main signaling pathways responsible for the pathogenesis of these diseases. HMGB1 is involved in the activation of innate and adaptive immunity, development of inflammation, and increased production of cytokines. In patients with rheumatoid arthritis, the production of HMGB1 and the number of cells secreting HMGB1 at specific inflammation areas are elevated [19, 20]. Experimental models of arthritis showed inhibitory effects of anti-HMGB1 antibodies on the development of synovial inflammation. HMGB1 is also found in SLE patients' plasma, and after being released from apoptotic cells, HMGB1 is bound to nucleosomes [21]. In turn, autoantibodies directed against nucleosomes and double-stranded DNA are characteristics for SLE. Our previous study demonstrated the expression of RAGE and HMGB1 in epithelial cells of sinonasal mucosa samples obtained from patients with chronic rhinosinusitis or in epithelial cells of middle ear cholesteatoma [12, 22]. We observed a strong correlation between the disease severity and RAGE expression.

Studies have shown that HMGB1 is involved in the pathogenesis of autoimmune thyroiditis and other chronic diseases by augmenting inflammation signaling and inflammatory infiltration [22–24]. Han et al. demonstrated that HMGB1 and its receptors are associated with the inflammatory mechanisms of GO and blocking of the HMGB1 pathway can be utilized to treat GO patients [25]. The authors demonstrated higher gene expression levels of RAGE and TLRs and higher mRNA and protein levels of HMGB1 in GO tissues compared to non-GO tissues. Moreover, blocking of HMGB1, RAGE, and TLR caused diminished production of proinflammatory cytokines confirming the participation of HMGB1 in GO inflammation. Plasma levels of HMGB1 were shown to correlate with the clinical activity score (CAS), indicating a valuable biomarker of disease activity. Being coherent with previous studies, we confirm the expression of RAGE and HMGB1 in GO tissues using immunohistochemistry. We observed a strong correlation between DON and RAGE expression in GO tissues. In addition, in GO tissues we demonstrated the presence of RAGE and HMGB1 positive inflammatory cells which were closely located to the vessels. We hypothesize that those cells may also play an important role in driving the inflammatory process. According to our knowledge, this is the first study that has shown the presence of RAGE and HMGB1 positive inflammatory cells in the GO adipose tissue microenvironment.

Peng et al. demonstrated that HMGB1 and RAGE expression on monocytes isolated from PBMCs was higher in patients with autoimmune thyroid diseases (AITD) compared to those isolated from healthy controls [26]. Their findings suggested a vital role of RAGE and HMGB1 in the pathogenesis of AITD. RAGE participates in inflammatory cell recruitment and chronic inflammation development, which are considered crucial aspects of GO pathogenesis [25, 27].

Pathogenesis of GO is closely associated with the presence of TRAb which involves autoantibodies directed against TSHR expressed not only on thyrocytes but also on orbital tissues. TRAb levels correlate with the clinical activity of GO [28]. Diana et al. reported a correlation between TRAb and the degree of ocular changes in GD [29]. However, the relationship between TRAb level and HMGB1 expression was not observed by us. In turn, Han et al. demonstrated a strong correlation between plasma level of HMGB1 and TRAb as well as CAS. Literature data report a correlation between RAGE levels in the serum and disease activity of other autoimmune diseases [30]. Studies analyzing the involvement of RAGE and HMGB1 in the pathogenesis of various inflammatory diseases are accompanied by experimental studies showing anti-RAGE/HMGB1 antibodies that prevent chronic inflammation [31, 32].

In summary, we demonstrated for the first time by using immunohistochemistry that RAGE and HMGB1 are overexpressed in GO patients in comparison with NC. We also observed a correlation between RAGE expression and occurrence of dysthyroid optic neuropathy (DON) or elevated levels of TSH Receptor Antibodies (TRAb) in GO patients. Overexpression of RAGE and HMGB1 might be associated with GO pathogenesis.

## Figures and Tables

**Table 1:** Clinical characterization of the Graves' ophthalmopathy (GO) patients included in this study at the day of surgery.

Characteristics	Patients (*n* = 23) 36 orbits	Normal controls (*n* = 15) 15 orbits
Sex (number of patients)		
Female	18	4
Male	5	11
Age (years)		
Range	35-73	36-56
Median	65	46
Disease status (number of orbits)		
Moderate-to-severe GO	14	
Severe GO		
With DON	12	
With severe keratitis	10	
Graves' disease (number of patients)	23	
Median CAS	2	
Thyroidectomy	7	
Median proptosis (mm)	23	
Median IOP (mmHg)	16	
Median BCVA	0.9	
Median fT4 (pmol/L)	19.4	
Median TRAb (U/L)	12.4	
Median aTPO (U/mL)	89.3	
Median aTG (U/mL)	144	

Abbreviations: DON: dysthyroid optic neuropathy; CAS: clinical activity score; IOP: intraocular pressure; BCVA: best-corrected visual acuity; fT4: free thyroxine; TRAb: anti-TSHR antibodies; aTPO: antithyroid peroxidase; aTG: antithyroglobulin.

## Data Availability

The research and clinical data used to support the findings of this study are included within the article.
